# Dynamic brain states in spatial neglect after stroke

**DOI:** 10.3389/fnsys.2023.1163147

**Published:** 2023-05-02

**Authors:** Sara Spadone, Francesco de Pasquale, Anna Digiovanni, Eleonora Grande, Luigi Pavone, Stefano L. Sensi, Giorgia Committeri, Antonello Baldassarre

**Affiliations:** ^1^Department of Neuroscience, Imaging and Clinical Sciences, University G. d'Annunzio of Chieti-Pescara, Chieti, Italy; ^2^Faculty of Veterinary Medicine, University of Teramo, Teramo, Italy; ^3^IRCCS NEUROMED, Pozzilli, Italy

**Keywords:** neglect, dynamic functional connectivity, brain states, modularity, cluster analysis

## Abstract

Previous studies indicated that spatial neglect is characterized by widespread alteration of resting-state functional connectivity and changes in the functional topology of large-scale brain systems. However, whether such network modulations exhibit temporal fluctuations related to spatial neglect is still largely unknown. This study investigated the association between brain states and spatial neglect after the onset of focal brain lesions. A cohort of right-hemisphere stroke patients (*n* = 20) underwent neuropsychological assessment of neglect as well as structural and resting-state functional MRI sessions within 2 weeks from stroke onset. Brain states were identified using dynamic functional connectivity as estimated by the sliding window approach followed by clustering of seven resting state networks. The networks included visual, dorsal attention, sensorimotor, cingulo-opercular, language, fronto-parietal, and default mode networks. The analyses on the whole cohort of patients, i.e., with and without neglect, identified two distinct brain states characterized by different degrees of brain modularity and system segregation. Compared to non-neglect patients, neglect subjects spent more time in less modular and segregated state characterized by weak intra-network coupling and sparse inter-network interactions. By contrast, patients without neglect dwelt mainly in more modular and segregated states, which displayed robust intra-network connectivity and anti-correlations among task-positive and task-negative systems. Notably, correlational analyses indicated that patients exhibiting more severe neglect spent more time and dwelt more often in the state featuring low brain modularity and system segregation and vice versa. Furthermore, separate analyses on neglect vs. non-neglect patients yielded two distinct brain states for each sub-cohort. A state featuring widespread strong connections within and between networks and low modularity and system segregation was detected only in the neglect group. Such a connectivity profile blurred the distinction among functional systems. Finally, a state exhibiting a clear separation among modules with strong positive intra-network and negative inter-network connectivity was found only in the non-neglect group. Overall, our results indicate that stroke yielding spatial attention deficits affects the time-varying properties of functional interactions among large-scale networks. These findings provide further insights into the pathophysiology of spatial neglect and its treatment.

## Introduction

Spatial neglect, a neuropsychological syndrome affecting around ~20–30% of all stroke patients (Buxbaum et al., 2004; Ringman et al., 2004), is characterized by an impairment in attending, processing, and responding to targets which are presented in the side of the space and body opposed to the brain lesion, which is more frequently in the right hemisphere (Halligan et al., 1989; Verdon et al., 2010; Corbetta and Shulman, 2011). This contralesional spatial bias is also associated with non-spatial deficits of sustained attention, arousal, and vigilance (Husain and Rorden, 2003).

Albeit investigated for a long-time, the neurofunctional correlates of spatial neglect are still debated (Husain and Rorden, 2003; Corbetta and Shulman, 2011; Bartolomeo et al., 2012; Karnath and Rorden, 2012). Lesion-to-symptom mapping studies have identified several brain structures related to neglect, such as inferior frontal (Husain and Kennard, 1996; Committeri et al., 2007; Corbetta et al., 2015), insular (Karnath et al., 2009; Corbetta et al., 2015), temporo-parietal (Karnath et al., 2001, 2004; Committeri et al., 2007; Corbetta et al., 2015) and inferior parietal (Mort et al., 2003) cortex, basal ganglia (Karnath et al., 2005; Corbetta et al., 2015), thalamus (Corbetta et al., 2015) as well as underlying white matter (Doricchi and Tomaiuolo, 2003; Karnath et al., 2009; Thiebaut de Schotten et al., 2014; Corbetta et al., 2015).

In recent years, such a challenge has been attempted within the framework of the so-called “connectomal diaschisis”, a novel type of diaschisis, which posits that a focal brain injury leads to widespread changes of large-scale networks among areas that are structurally spared and distant from the lesion site (Carrera and Tononi, 2014) (for reviews on stroke, see Varsou et al., 2014; Baldassarre et al., 2016; Siegel et al., 2022). Indeed, two pioneer studies showed that the extent of the rightward bias in neglect patients is associated with a breakdown of the inter-hemispheric resting-state functional connectivity (FC) MRI among intact fronto-parietal areas of the dorsal attention network that is involved in the control of visuo-spatial attention (He et al., 2007; Carter et al., 2010). Subsequently, in our previous work (Baldassarre et al., 2014), we detected two large-scale patterns of abnormal functional connectivity associated with the severity of spatial neglect in a large cohort of acute stroke patients: reduction of inter-hemispheric FC within dorsal attention/sensory motor networks as well as loss of negative FC (i.e., anti-correlation) between these networks and the default mode network. More recently, by adopting a graph-theoretic approach, in two companion studies, we have shown that spatial neglect is characterized by widespread changes in the brain topological organization at different scales of network analysis (de Pasquale et al., 2021a; Spadone et al., 2022). At the micro-scale level, we identified two sets of neglect-relevant hubs derived using the betweenness centrality metric [i.e., the number of the shortest paths passing through a given node (Rubinov and Sporns, 2010; de Pasquale et al., 2021a)]. Specifically, one group of neglect hubs was detected in higher-order associative systems, such as the dorsal and ventral attention, frontoparietal, and default mode networks. These hubs exhibited lower centrality as well as higher shortest paths length (i.e., less efficient) associated with severe neglect. Conversely, a reverse pattern was observed in a second cohort of neglect hubs dislocated in lower-level sensory-processing systems such as the visual and motor networks. At meso-scale level, neglect was associated with a loss of system segregation, i.e., the balance between the functional specialization and dynamic integration of distinct and segregated (sub)networks (Tononi et al., 1994; Wig, 2017), involving higher-order associative networks such as dorsal attention, fronto-parietal and default mode as well as the sensorimotor network (Spadone et al., 2022).

Overall, these lines of evidence indicate that neglect is characterized by widespread alteration of resting-state networks as well topological changes in the brain, suggesting a maladaptive shift from higher-order to low-level sensory-processing systems.

However, the brain is a dynamic system characterized by transient states with different degrees of integration and segregation among multiple large-scale networks (de Pasquale et al., 2021a,b). Notably, recent functional MRI studies adopting a dynamic functional connectivity approach have identified time-varying properties of functional connections among brain networks (Calhoun et al., 2014). Clinically, several reports indicated that such brain states are affected after stroke (Bonkhoff et al., 2020, 2021a,b; Wang et al., 2020; Favaretto et al., 2022). Hence, the dynamic connectivity method can capture transient conditions of network reconfigurations as they happen after a focal brain lesion. Therefore, the goal of the current study was to investigate whether the above-described network modulations exhibit temporal variations which can be potentially related to spatial neglect. To this aim, we estimated functional connectivity dynamics (Calhoun et al., 2014) on our previously collected dataset (de Pasquale et al., 2021a; Spadone et al., 2022) to characterize the temporal fluctuations of brain states associated with spatial neglect after right hemisphere strokes. Since neglect has been associated with changes of functional connectivity in multiple large-scale networks, we expect to identify brain states characterized by widespread alterations of their functional architecture.

## Methods

### Stroke patients and assessment of neglect

A cohort of twenty right-hemisphere damaged patients (mean age 65.1 y, SD = 12.3 y) was enrolled within 2 weeks since first-time stroke onset. The Inclusion criteria were as follows: (1) Clinical diagnosis of right hemisphere stroke (ischemic or hemorrhagic) at hospital discharge; (2) Persistent stroke symptom(s) at hospital discharge; (3) Awake, alert, and able to complete study tasks; (4) Age > 18. Exclusion criteria: (1) Severe psychiatric or neurological disorders/conditions; (2) Claustrophobia; (3) Body metal not allowing 3T MRI. Table 1 displays the demographic and clinical information of stroke patients. The Bells Cancellation Test and Letter Cancellation Test assessed the severity of visual neglect. Patients were classified as having neglect if their Center of Cancellation (CoC) (Binder et al., 1992) score was above the normative cut-off in at least one test, 0.081 and 0.083, respectively (Rorden and Karnath, 2010) (Table 1 displays demographic and clinical information of the cohort of patients).

**Table 1:** Demographic and clinical characteristics of the stroke patients.

**ID**	**Age at stroke (y)**	**Sex**	**Education (y)**	**Time post-stroke (y)**	**Neglect**	**Lesion type**	**Lesion site**
4	69	F	8	3	+	I	Cau; Pal; Pu; STG
8	92	F	5	2	+	I	Cau; Pal; Put; Ins; IntCap; ExtCap
9	67	M	13	7	+	I	Cau; Pal; Put;
11	65	F	8	14	+	H	Pal; Put; Ins; ExtCap; STG
14	60	M	13	2	+	I	Tha
21	73	F	5	5	+	I	Put; IntCap
22	53	M	8	10	+	H	Pal; Put
24	74	F	5	5	+	I	Put; Ins; Cau; CorRad; IntCap
31	56	M	13	4	+	I	Tha
32	73	F	5	11	+	I	IFG; Ins; Put; ExtCap
33	76	F	5	7	+	I	Put; Ins; STG; IFG; CorRad; IntCap
3	84	F	8	7	–	I	BS
6	73	M	13	5	–	H	PHG; LG
7	41	M	13	14	–	I	SPL; PreCun; AG; SLF
16	62	M	13	5	–	I	Tha
20	65	M	13	8	–	I	LOG; FFG; PHG
23	77	F	5	14	–	I	MFG; PrCG; SPL
26	73	M	5	12	–	I	CorRad
30	62	F	8	4	–	I	SLF
34	51	M	8	9	–	I	Put; Cau; CorRad; IntCap; SLF

Y, year; M, male; F, female; +, presence of neglect; –, absence of neglect; I, ischemic; H, hemorrhagic; PHG, Parahippocampal Gyrus; Put, Putamen; Cau, Caudate; Pal, Pallidum; BS, Brain Stem; STG, Superior Temporal Gyrus; Lingual Gyrus; SPL, Superior Parietal Lobule; PreCun, Precuneus; AG, Angular Gyrus; SLF, Superior Longitudinal Fasciculus; Ins, Insula; Tha, Thalamus; LOG, Lateral Occipital Gyrus; FFG, Fusiform Gyrus; IntCap, Internal capsule; ExtCap, External Capsule; MFG, Middle Frontal Gyrus; PrCG, Pre-Central Gyrus; CorRad, Corona Radiata; IFG, Inferior Frontal Gyrus.

### Functional MRI acquisition

MRI scanning was performed with a GE Signa HDxt 3T at the IRCCS NEUROMED (Pozzilli, Italy) within 24 h of the neuropsychological assessment. Structural scans consisted of: (1) an axial T1-weighted 3D SPGR (TR = 1,644 ms, TE = 2.856 ms, flip angle = 13 deg, voxel size = 1.0 × 1.0 × 1.0 mm) and (2) an axial T2-weighted turbo spin-echo (TR = 2.856 ms, TE = 127.712 ms, slice thickness 3 mm, matrix size: 512 × 512). Resting-state functional scans were acquired with a gradient echo EPI sequence with TR = 1,714 ms, TE = 30 ms, 34 contiguous 3.6 mm slices, during which participants were instructed to keep open eyes in a low luminance environment. Three resting-state fMRI runs of 7.5 min were collected.

### Lesion segmentation

The lesions were manually segmented using MRIcron software (www.mayo.edu) by examining T1-weighted and T2-weighted images simultaneously displayed in the atlas space. All segmentations were reviewed by a trained radiologist of NEUROMED (GG in de Pasquale et al., 2021a).

### fMRI data pre-processing

Functional data were pre-processed in CONN toolbox (https://www.nitrc.org/projects/conn/; Whitfield-Gabrieli and Nieto-Castanon, 2012) by employing the default pre-processing pipeline (Nieto-Castanon, 2020) which included the steps of functional realignment and unwarping, slice-timing correction, potential outlier scans identification, direct segmentation and normalization in Montreal Neurological Institute (MNI) space and smoothing with a 6-mm kernel. Head-motion contaminated frames were identified through the global BOLD signal and the amount of patient-motion. Specifically, all the functional volumes in which the global BOLD signal changes was above 5 SD or the framewise displacement was above 0.9 mm were classified as outliers and then employed as confounding regressors of non-interest to remove their influence on the BOLD signal timeseries. Furthermore, pre-processed functional data underwent the CONN's default denoising pipeline to estimate and regress out physiological and other noise sources. Specifically, an anatomical component-based noise correction procedure (aCompCor) (Behzadi et al., 2007) was employed to identify and remove physiological noise from white matter and cerebrospinal fluid, subject-motion parameters (Friston et al., 1995), and outlier scans (Power et al., 2014). Next, based on previous dynamic functional connectivity MRI studies (Leonardi and Van De Ville, 2015), a temporal band-pass filter of 0.029–0.15 Hz was applied to the time series. Overall, several denoising steps, including CompCor correction, outlier censoring, motion regression, and linear detrending, were computed simultaneously before the band-pass filtering. Finally, the residual BOLD time-series for each region of interest were employed for estimating the dynamic brain states.

### Resting-state networks

In the current study, we employed a functional brain parcellation implemented in CONN toolbox that includes a set of 30 regions of interest (ROIs) defined from CONN's Independent Component Analyses of Human Connectome Project dataset (497 subjects) (Whitfield-Gabrieli and Nieto-Castanon, 2012; Nieto-Castanon, 2020). Specifically, the ROIs belonged to seven resting state networks comprising visual, dorsal attention, sensorimotor, cingulo-opercular, language, fronto-parietal, and default mode networks (Supplementary Table 1).

### Brain state analysis in the whole cohort of patients

#### Brain states identification

To estimate the dynamic functional connectivity, the time course of the BOLD signal of the 260 volumes (in all participants) was segmented into 34-s (20 TRs) sliding windows (see recommendation by Leonardi and Van De Ville, 2015), moving the onset every 1.7 s (1 TR), for a total of 241 sliding windows. Next, for each sliding window, the functional connectivity was obtained through the Pearson correlation coefficient (*r*) among fMRI signals of all the possible pairs of the 30 parcellation nodes. To obtain normally distributed values, *r* scores were Fisher-transformed into *z*-scores. The output of this analysis is a temporal series of FC matrices. To identify a set of states representing the most recurrent connectivity patterns over time, we ran a K-means clustering. Specifically, the clustering algorithm was applied to the set of windowed FC matrices of all subjects concatenated along the time dimension resulting in 241^*^20 = 4,820 FC patterns. The clustering algorithm was implemented using the Manhattan (cityblock) as the distance among the 4,820 observations.

To estimate the optimal number of clusters, we ran the clustering algorithm for different values of classes. For each output, we computed a mixed performance criterion (MPFC, see Spadone et al., 2012; de Pasquale et al., 2021b) which is the product of different clustering performance criteria:


$$\begin{array}{l} MPFC=\;\frac{CS^{*}AS^{*}DI}{DB}, \end{array}$$

where CS is the average cluster size, AS is the average silhouette, DI is the Dunn Index, and DB is the Davies Bouldin index. In this way, several aspects can be combined and considered in the cluster number estimation. A detailed discussion on these parameters can be found in Spadone et al. (2012). The optimal number of clusters corresponds to the peak of the MPFC. The centroid of each cluster putatively reflects a connectivity “state”. These analyses were performed using in-house developed software in MATLAB (2022, Natick, Massachusetts: The MathWorks Inc.).

#### Brain states characterization

To characterize the identified brain states, we investigated their internal arrangement into modules or communities (Medaglia, 2017). To this aim, we computed on the centroid matrix of each state in each individual patient, the brain modularity, and the system segregation. These two indices are behaviorally relevant for spatial neglect (Siegel et al., 2018; Spadone et al., 2022). Specifically, brain modularity represents a measure of the goodness of network subdivision into well-defined modules or communities (Bullmore and Sporns, 2009). Such a score was estimated by employing the Louvain modularity algorithm implemented in the brain connectivity toolbox (Rubinov and Sporns, 2010). This procedure yielded for each patient a brain modularity value associated with each brain state. Moreover, we computed the system segregation, a measure that captures the balance between functional specialization and dynamic integration of distinct and segregated (sub)networks (Tononi et al., 1994; Wig, 2017). In detail, the system segregation was computed as described in Chan et al. (2014): for each patient and each of the seven resting state networks, the within-network FC (WNFC) and the between-network FC (BNFC) were computed for each of the seven resting state networks. Specifically, for each centroid matrix of each state, WNFC was derived as the mean correlation, among all possible pairs of regions within that network, whereas BNFC as the averaged correlation among regions of a given network and all other nodes of the rest of the brain connectome. This computation produced seven values (one for each network) that were then averaged to obtain the system segregation score. As for the estimation of the modularity, this analysis returned for each patient a system segregation value for each brain state.

#### Temporal dynamics of brain states

Furthermore, we computed two dynamic connectivity measures: the fraction time (the percentage of the total time a subject spent in a given connectivity state) and the dwell time (the time a subject spent in a state without switching to another one) for each of the states. Furthermore, we investigated the differences in time-varying properties of the identified brain states among neglect and non-neglect groups. To this aim, for each brain state, we carried out a two-sample (i.e., neglect vs. non-neglect) *t*-test on fraction times and dwell times. Finally, to examine the link between neglect severity and the temporal dynamics of brain states, we computed a set of Spearman rank correlations between the averaged CoC scores of Bells and Letter tests with the fraction times and dwell times of each brain state.

### Brain state analysis in the sub-cohorts of patients with and without neglect

Finally, to study neglect-specific brain states, we separately extracted them from the sub-cohorts of patients with and without neglect by employing the above-described pipeline. Specifically, we computed the K-means clustering on the patient's dynamic functional connectivity matrices by grouping them in two distinct sub-groups (neglect, *n* = 11/non-neglect, *n* = 9). Of note, compared to the main analysis, this procedure yielded two sets of centroid matrices obtained with the unique contribution of the two sub-cohorts. Next, we computed the modularity and system segregation based on these centroid matrices. Finally, fraction times and dwell times were also extracted.

## Results

### Behavior and lesion topography

As reported in our previous studies (de Pasquale et al., 2021a; Spadone et al., 2022), 11 patients (55%) were classified as neglect since they scored above the CoC cut-off at least in one cancellation test (Rorden and Karnath, 2010). Moreover, within the neglect sub-group, some patients also exhibited deficits in general cognitive efficiency (60%), executive functions (57%), praxis abilities (37%), and verbal memory (66%). Finally, as previously described, the spatial topography of lesion distribution indicated that the highest incidence of strokes was present in the middle cerebral artery territory, with the thalamus and putamen as the most frequently damaged regions.

### Static functional connectivity

Before estimating the dynamic functional connectivity, we computed the brain modularity and system segregation based on the average of the dynamic functional connectivity matrices. The analyses revealed that neglect patients exhibited lower static system segregation (mean = 0.5085, SD = 0.2071) as compared to non-neglect patients (mean = 0.6726, SD = 0.1020) [*t*_(18)_ = −2.1646, *p* = 0.0441]. Furthermore, no differences in terms of brain modularity were detected among two sub-groups [neglect, mean = 0.8211, SD = 0.4712; non-neglect, mean = 0.9869, SD = 0.4945; *t*_(18)_ = −0.7656, *p* = 0.4538].

### Brain states in the whole cohort of patients

**Figure 1:**
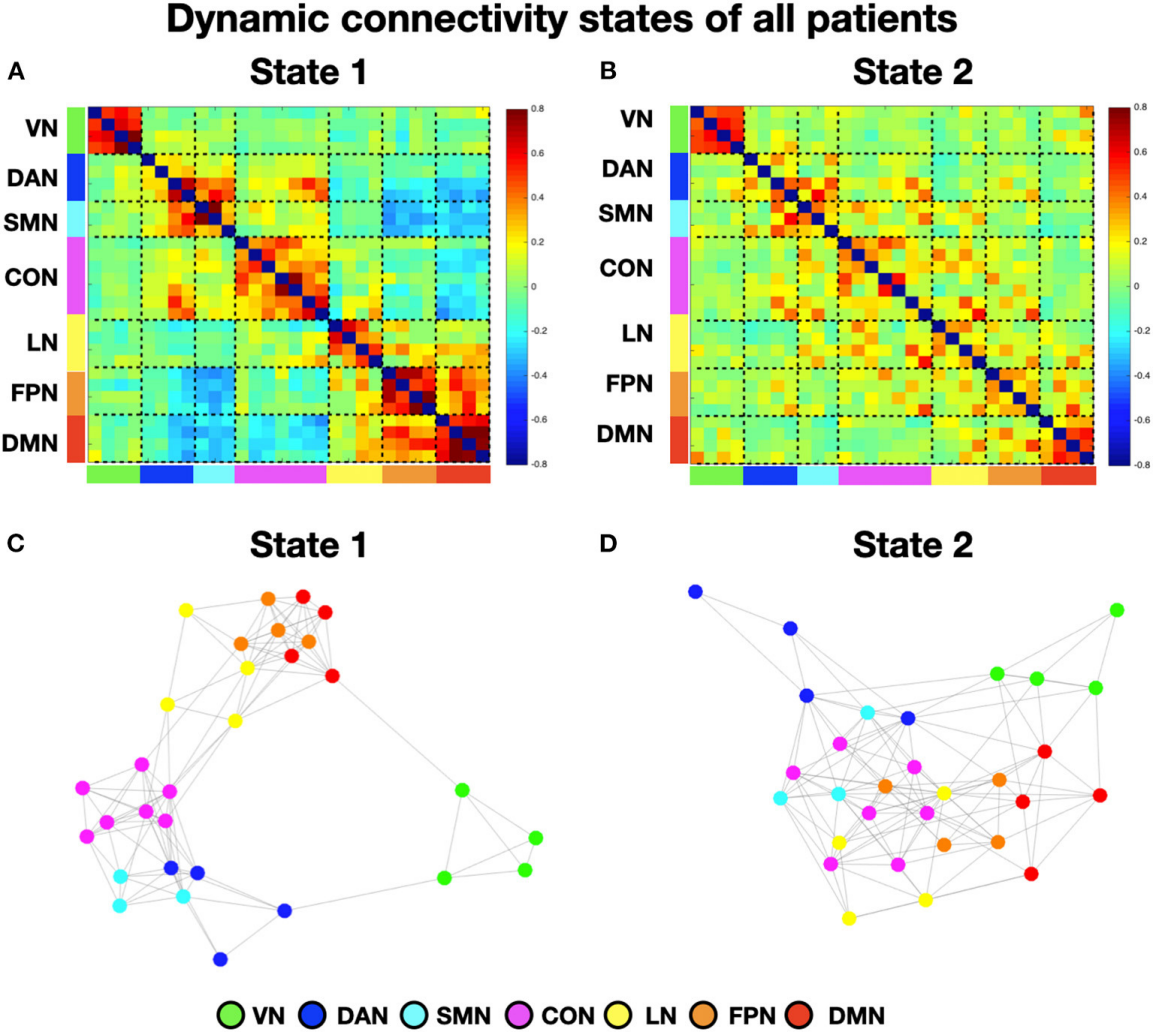
Dynamic connectivity states of all patients. **(A, B)** Display the centroid of the functional network connectivity states identified in the whole cohort (i.e., with and without neglect) of patients. The color bar indicates the Fisher-transformed *z*-scores of the Pearson correlation coefficient (*r*) among fMRI signals between all the possible pairs of nodes. **(C, D)** Show the spring-embedded representation of the centroid of the functional network connectivity states. VN, visual network; DAN, dorsal attention network; SMN, sensory-motor network; CON, cingulo-opercular network; LN, language network; FPN, fronto-parietal network; DMN, default mode network.

To perform the k-means analysis on the entire sample, i.e., patients with and without neglect, we first estimated the number of optimal classes through MPFC. We observed a clear MPFC peak corresponding to two clusters. Thus, we run K-means and we identified two distinct functional connectivity states, i.e., brain states, reoccurring during the functional MRI scans. Specifically, brain state 1 (43.8% of occurrence) was characterized by robust positive connectivity within each network (see structures around the diagonal of the reported matrix in Figure 1A). We observed a strong interaction, i.e., positive coupling, among two sets of systems comprising dorsal attention, sensorimotor, and cingulo-opercular networks as well as language, fronto-parietal, and default mode networks, respectively (Figure 1A). Finally, state 1 exhibited strong anti-coupling, i.e., negative inter-networks connectivity (i.e., anti-correlations), among two groups of networks: dorsal attention, sensorimotor, and cingulo-opercular on one side, vs. fronto-parietal and default mode (Figure 1A). By contrast, state 2 (56.2% of occurrence) featured weaker intra- and inter-network positive connections and a neglectable inter-network negative connectivity (Figure 1B). In state 2, apart from the visual, sensorimotor, and default mode network, the other networks lose their internal coupling and show sparse connections with the rest of the brain.

Next, to characterize the organization of brain networks of the two states, we computed the brain modularity and system segregation indices (see Section Methods). A two-tails paired *t*-test indicated that state 1 exhibited higher modularity (mean = 1.44; SD = 0.81) as compared to state 2 (mean = 0.65; SD = 0.33) (*t* = 4.6; *p* < 0.0005). Similarly, state 1 exhibited higher system segregation (mean = 0.6439; SD = 0.163) as compared to state 2 (mean = 0.5; SD = 0.2) (*t* = 4.53; *p* = 0.0006). Taken together, these results indicate that in state 1, as compared to state 2, network communities are more clearly differentiated (see Figures 1C, D for the spring-embedded representation of the centroid graph of state 1 and state 2, respectively). We note a strong interaction between default mode and fronto-parietal networks (see Figure 1C).

**Figure 2:**
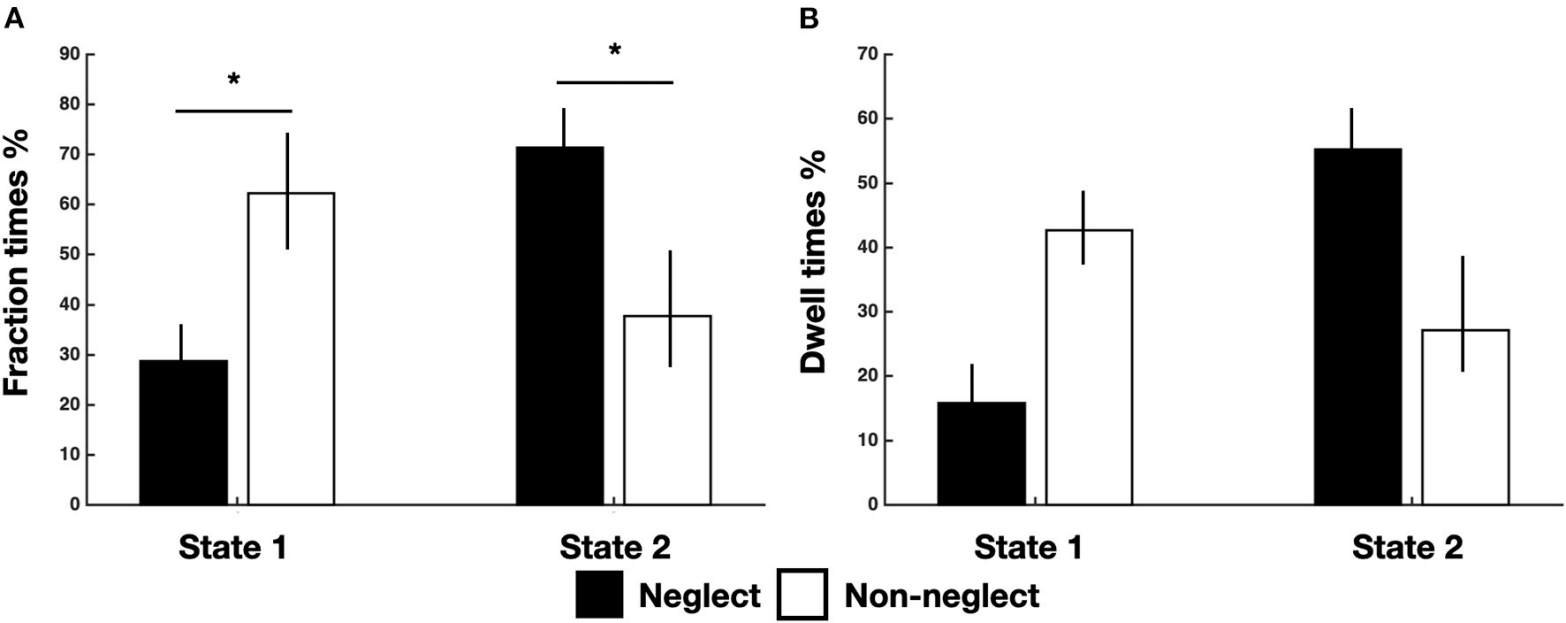
Temporal properties of brain states for neglect and non-neglect patients. Bar graphs indicate fraction **(A)** and dwell **(B)** times of each state for neglect (black) and non-neglect (white) patients, respectively. *p < 0.05, FDR-corrected.

Successively, we investigated the differences in time-varying properties of two brain states among neglect and non-neglect groups. A set of two-sample *t*-tests on fraction times of state 1 and state 2 (high/low modularity and system segregation) indicated that neglect patients, as compared to non-neglect patients, exhibited lower and higher fraction times in state 1 (*t* = −2.68, *p* = 0.036, FDR-corrected) and in state 2 (*t* = 2.68, *p* = 0.036, FDR-corrected), respectively (Figure 2A). Moreover, two-sample *t*-tests on dwell times showed two marginally significant trends: in state 1 neglect group displayed lower dwell times as compared to non-neglect (*p* = 0.08, FDR-corrected); in state 2 it was observed a reverse pattern (*p* = 0.09, FDR-corrected) (Figure 2B). Taken together, these results indicate that neglect patients, compared to non-neglect ones, are generally less involved in state 1. In fact, they spend more continuous time and dwelt more in state 2 (vs. state 1).

### Association between neglect severity and temporal dynamics of brain states

**Figure 3:**
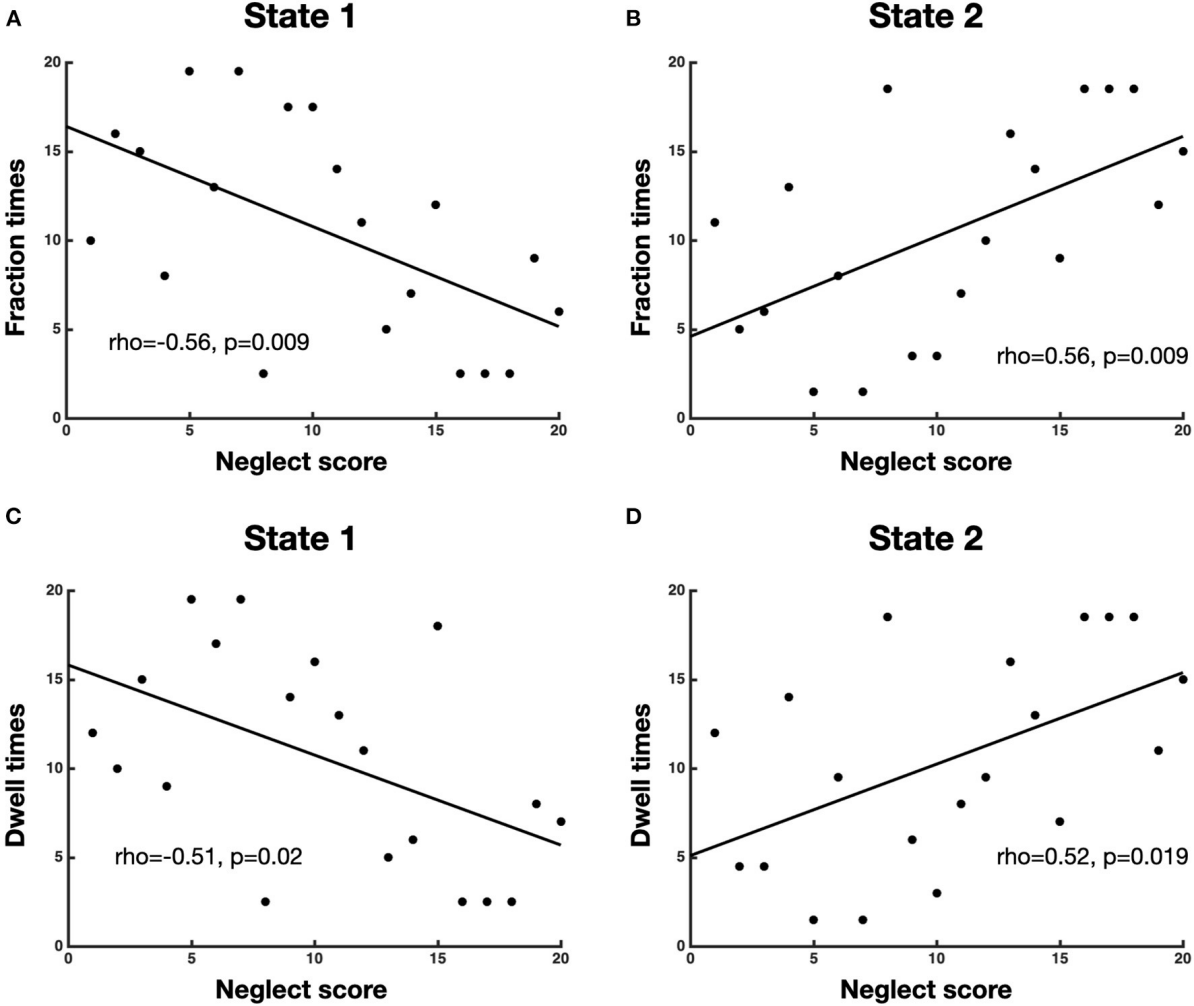
Association between neglect severity and temporal dynamics of brain states. The scatterplots display the Spearman rank correlation between neglect score and fraction times of state 1 **(A)** and state 2 **(B)** as well as dwell times of state 1 **(C)** and state 2 **(D)**. Each dot represents a patient (*n* = 20). To be noted, high value on x-axis (neglect score) means severe neglect and vice versa.

To investigate the association between neglect severity and the temporal dynamics of brain states, we employed the Spearman rank test to correlate the averaged CoC scores of Bells test and Letter test with the fraction times and dwell times of each brain state. The analyses revealed that neglect score was negatively correlated with fraction times of state 1 (high modularity and system segregation) (*r* = −0.56, *p* = 0.009, FDR-corrected) such that patients with severe neglect (high score) spent less amount of time in such state and vice versa (Figure 3A). By contrast, the neglect measure exhibited positive correlation with the fraction times of state 2 (low modularity and system segregation) (*r* = 0.56, *p* = 0.009, FDR-corrected), indicating that patients with severe neglect (high score) spent more amount of time in that state and vice versa (Figure 3B). On the same line, it was detected that neglect score was negatively correlated with dwell times in state 1 (*r* = −0.51, *p* = 0.022) as well as positively correlated with dwell times in state 2 (*r* = −0.52, *p* = 0.019). These associations indicate that more impaired patients dwelt less and more often in state 1 and state 2, respectively (Figures 3C, D). Overall, these findings show that patients exhibiting stronger rightward bias (i.e., more severe neglect) spent more time and dwelt more often in the state featuring low brain modularity and system segregation and vice versa.

### Brain states in the sub-cohorts of patients with and without neglect

**Figure 4:**
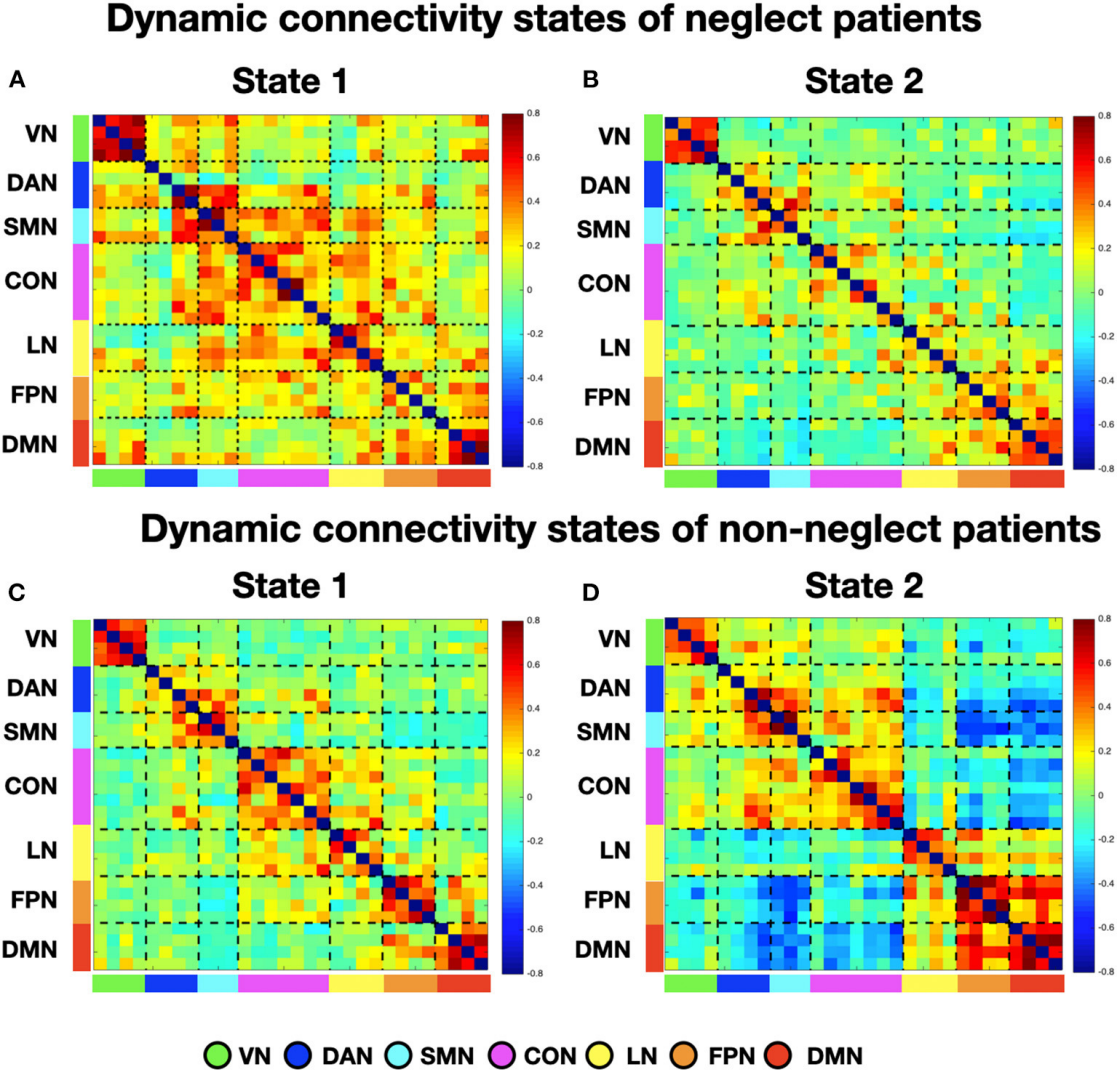
Dynamic connectivity states of neglect and non-neglect patients. **(A, B)** Display the centroid of the functional network connectivity states identified in the sub-cohort of neglect patients, whereas **(C, D)** indicate those of non-neglect patients. Color bar and abbreviations as in Figure 1.

**Figure 5:**
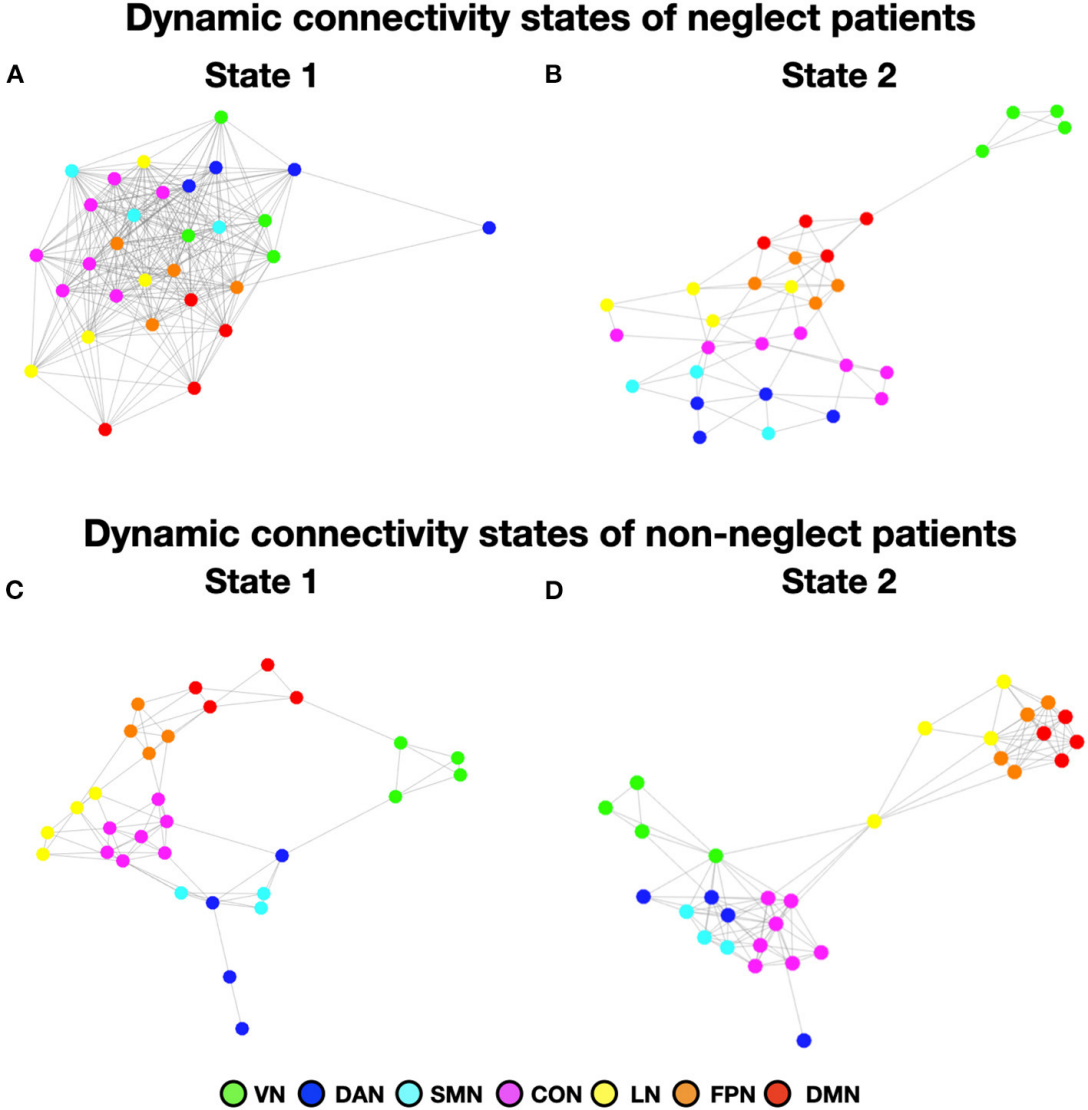
Dynamic connectivity states of neglect and non-neglect patients. **(A, B)** Show the spring-embedded representation of the centroid of the functional network connectivity states identified in the sub-cohort of neglect patients. In contrast, **(C, D)** refer to those of non-neglect patients. Abbreviations as in Figure 1.

To investigate neglect-specific aspects of the brain states, we extracted again them from each sub-cohort separately (see Section Methods). For the neglect cohort, we identified two brain states characterized by distinct connectivity profiles. Specifically, state 1 (28.5% of occurrence) featured widespread high positive functional connections both within and between networks (Figure 4A). Hence, such connectivity pattern blurred the distinction among functional systems. This state was not detected in the whole sample analysis. Furthermore, state 2 (71.5% of occurrence) was characterized by modest intra-network connections as well as exclusively positive values of inter-network connectivity (Figure 4B). Of note, the network connectivity configuration of this state resembled the one of state 2 detected in the whole cohort of patients (Figure 1B). The analyses in the non-neglect group also identified two brain states. In detail, state 1 (62.2% of occurrence) featured robust positive intra-network connectivity as well as interactions between networks (Figure 4C). Moreover, state 2 (37.8% of occurrence) (Figure 4D) exhibited a connectivity profile that strongly recapitulates that one of state 1 (i.e., positive intra-network and negative between-networks connectivity) identified in the whole sample (Figure 1A). Next, as in the whole sample analyses, we investigated the network configurations of the four states by computing and comparing the brain modularity and system segregation metrics (see Section Methods) with the caveat that the sample size of the sub-cohorts is relatively small (see Figure 5 for the spring-embedded representation of the centroid graph of state 1 and state 2 in the 2 sub-cohorts). A set of paired and two-sample *t*-tests on the modularity values indicated that state 1 of neglect group exhibited lower modularity as compared to state 2 of neglect group (*t* = −5.83; *p* = 0.01) as well as state 1 (*t* = −2.66; *p* = 0.02) and state 2 (*t* = −10.2; *p* = 0.000005) of non-neglect group. Furthermore, it was observed that state 2 of non-neglect group showed higher modularity as compared to state 1 of non-neglect group (*t* = 7.28; *p* = 0.0003) as well as state 1 (*t* = 10.2; *p* = 0.000005) and state 2 (*t* = 4.66; *p* = 0.0003) of neglect group. The same set of analyses on the system segregation indicated that state 2 of the non-neglect group exhibited a higher score than state 1 (*t* = 6.05; *p* = 0.0001) of the neglect group.

To summarize, the analyses on the sub-groups of patients with and without neglect indicated that: (i) a state featuring the lowest degree of modularity and system segregation, with blurred separation among networks, was detected solely in the sub-group of neglect patients (Figures 4A, 5A). Notably, this configuration was not highlighted by the analysis in the whole sample of patients; (ii) two states with comparable connectivity profiles were observed one in each sub-cohort (Figures 4B, 5B for neglect; Figures 4C, 5C for non-neglect); (iii) a highly modular and segregated state showing a clear distinction among sub-systems as well as robust negative connectivity between task-positive and task-negative systems was described only in the non-neglect group (Figures 4D, 5D).

### Control analyses

We carried out a set of control analyses to assess whether the differences in terms of brain states among two sub-groups of patients were related to variables of interests. Specifically, we compared the overall NIHSS scores, the lesion size, and the number of outlier scans between neglect and non-neglect patients, by means of two-tail two-sample *t*-tests. Regarding the NIHSS, the overall symptom severity of neglect group (mean = 11.6, SD = 5.33, *n* = 10 since for one patient the score was not available) was not different as compared to the one of non-neglect group (mean = 8.5, SD = 5.95, *n* = 8 since for one patient the score was not available) (*t* = 1.16, *p* = 0.26). Furthermore, the lesion size of neglect group (mean = 9.98 cm^3^; SD = 9.23 cm^3^; *n* = 11) and non-neglect group (mean = 8.95 cm^3^; SD = 16.51 cm^3^; *n* = 9) did not differ (*t* = 0.1762, *p* = 0.862). Finally, no differences were observed between groups (*t* = 1.49, *p* = 0.159) in terms of outlier scans. Taken together, these analyses indicate that the overall symptom severity, the amount of structural damage as well as the head movements do not account for the association between spatial neglect and patterns of dynamic functional connectivity.

## Discussion

In the current study, we investigated the brain states associated with the pathology of spatial neglect in a cohort of acute right-hemisphere damaged patients. To this aim, we estimated the dynamic functional connectivity MRI which allows us to assess brain network variations in a time-scale resolution of seconds (Allen et al., 2014; Calhoun et al., 2014). By employing the sliding window approach and clustering analysis we identified two brain states featuring distinct connectivity profiles characterized by the degree of brain modularity and system segregation. Specifically, we observed that neglect, compared to non-neglect patients, spent more time in a low modularity and segregation state, characterized by weak intra-module connections and widespread positive interactions among modules. By contrast, non-neglect patients occupy larger fractions of time in high modularity and segregation states comprising high within-network functional connections, sparse between-networks interactions, and anti-correlation between the so-called task-positive and task-negative systems. Finally, a state with robust intra- and inter-network connectivity, low modularity and system segregation was detected exclusively in the neglect sub-group.

Brain modularity and system segregation represent key features of the mesoscale organization of the functional architecture of the brain, which orchestrate the processing of information among multiple networks (Bullmore and Sporns, 2009; Medaglia, 2017; Wig, 2017). The former indexes the extent to which a network can be subdivided into clearly distinct and non-overlapping communities or sub-systems. The latter quantifies the balance of intra-network integration and between-networks segregation. Our results clearly indicate that neglect patients exhibit a preference for brain states in which the distinctions among functional sub-systems are less defined or even blurred. Such brain configurations might represent a maladaptive response to a brain insult, i.e., focal lesion, such as a dedifferentiation-like mechanism (Fornito et al., 2015) characterized by the loss of the physiological balance between excitation and inhibition within neural systems. In this scenario, impaired behavior, e.g., visuo-spatial attention deficit, would be mediated by activations of task-irrelevant brain areas and by interactions among multiple functional systems that are not usually related to such behavior. Therefore, such pattern would result into the reduction of the network specialization (Li et al., 2001). This interpretation is consistent with several observations described in prior neuroimaging studies in neglect patients, both acutely and longitudinally. First, the rightward bias has been associated with the hyper- and hypo-activations of the left (contra-lesional) and right (ipsilesional) dorsal fronto-parietal attention regions, respectively (He et al., 2007). Such inter-hemispheric functional imbalance would result from affected excitatory and inhibitory mechanisms among the two hemispheres (Friedland and Weinstein, 1977; Kinsbourne, 1977). Notably, this pattern resolves over time as the recovery takes place. Second, the degree of spatial and non-spatial deficits in neglect has been linked, both acutely and longitudinally, to a loss of negative functional connectivity (i.e., segregation) between the dorsal attention and default mode networks in the right hemisphere (Baldassarre et al., 2014). Once again, a restoration of this pattern occurred in parallel with recovery (Ramsey et al., 2016). Finally, a neglect-relevant reduction of static system segregation of multiple large-scale networks at the acute stage (Spadone et al., 2022), as well as a restoration of brain modularity alongside the spontaneous recovery, has been reported (Siegel et al., 2018). Converging lines of evidence indicate that the degree of brain modularity and system segregation is relevant for the functional brain organization during lifespan in health and diseases (Chan et al., 2014; Marek et al., 2015; Ewers et al., 2021).

An important question is whether brain states featuring low modularity and system segregation might represent a key feature of the brain functional organization in other neurological conditions after focal lesions. Overall, current results generally agree with the findings obtained by recent studies that investigated brain states in stroke cohorts by employing a similar approach to that adopted in our work. Favaretto et al. (2022) identified five brain states characterized by different degrees of modularity as well as anti-correlation between dorsal attention and default mode networks in a large cohort of stroke patients. Crucially, the authors observed a preference of patients toward two states characterized by a high degree of integration among multiple networks and relatively high positive dorsal attention-default mode connectivity. This is in line with what we described here as state 2 of the whole cohort. Similarly, Wang et al. (2020) described four brain states and showed that patients with pontine stroke, compared to healthy controls, spent larger fraction times in a state featuring low segregation between networks as well as less fraction times in a state characterized by high segregation and anti-correlations among default mode network and task-positive systems. Notably, these two network configurations are very similar to the ones of our state 1 and state 2, respectively. In a longitudinal study, Bonkhoff et al. (2021b) identified three brain states: state 1 exhibited the highest segregation, with highly positive intra-domain connectivity, negative connectivity of visual network with somatomotor and cognitive networks; state 2 showed weak positive connectivity within network and near zero inter-networks connections; state 3 featured a network configuration in between state 1 and state 3. Critically, it was observed that more severely affected patients spent more time in state 1, i.e., a highly segregated state. In another recent fMRI study in the motor domain by Bonkhoff et al. (2020), the authors investigated in a cohort of acute stroke patients the association between upper limb deficit and brain states derived from the dynamic functional connectivity of three regional domains of the motor system, namely, cortical, subcortical, and cerebellar. Notably, they showed that severely affected patients exhibited a preference for a brain state characterized by high positive connections within each domain as well as anti-correlations among regions of different modules, hence featuring high level of segregation. This set of results is in contrast with those described in our study as neglect patients spent more time in a state with low modularity and system segregation and without (or reduced) inter-networks anti-correlations. However, it has been proposed that both extremes of either low or high levels of modularity and system segregation, i.e., inverted U-shaped pattern, lead to maladaptive behavior (Wig, 2017). While increased connectivity among systems can generate a dedifferentiated state, a network configuration characterized by robust segregation would result in a loss of interactions among areas of different systems or even in a disconnected state. Hence, in such a scenario, other systems might not support isolated communities under attack. Moreover, while the latter study (Bonkhoff et al., 2020) focused on the sub-domains of the motor network, here we employed a whole-brain functional parcellation comprising seven large-scale networks. The difference in the granularity level makes the comparisons of the two results difficult. It also does not exclude the possibility that brain states derived from sub-components of neglect-relevant systems, e.g., parietal and frontal areas of the dorsal attention network, might feature in highly segregated configuration. Moreover, in their longitudinal study (Bonkhoff et al., 2021b), the authors estimated dynamic FC on independent components, which might not be directly comparable with time series of brain regions identified by an atlas. Finally, the parcellation used by Bonkhoff et al. emphasized visual and sensorimotor areas rather than association areas (i.e., yielded larger number of components).

Beside the above-described aspects, a key methodological point refers to the choice of the approach used for the identification of brain states. Many approaches have been developed to estimate dynamic functional connectivity, and among them the most popular one is the sliding window method which is based on the partitioning of the time-series into overlapped temporal segments and the calculation of the functional connectivity between two ROIs for each window (Hutchison et al., 2013a; Calhoun et al., 2014). The concept of functional connectivity is wide and includes any kind of statistical relationship between time series. A largely used approach to measure windowed correlation in resting-state fMRI research is Pearson correlation coefficient (Hutchison et al., 2013b; Zalesky et al., 2014; Kaiser et al., 2016; Spadone et al., 2021). Another approach employed for estimating dynamic correlation is the sparse inverse covariance matrix (Allen et al., 2014; Damaraju et al., 2014). Among these methods, in the current study we estimated Pearson's correlation. As compared to other possible metrics, Pearson's correlation exhibits several advantages: (i) it requires less computational time; (ii) it is not dependent on the choice of the regularization parameter used to introduce spatial sparsity, which is currently discussed in the literature (see Zhang et al., 2021); (iii) it allows the comparison with our previous works on static functional connectivity in stroke patients (Baldassarre et al., 2014; de Pasquale et al., 2021a; Spadone et al., 2022). However, a common criticism of the Pearson correlation is its sensitivity to indirect functional relations between pairs of regions that are mediated by a third region. Notably, sparse representation approach is employed to overcome this issue, yielding a measure of direct interactions by removing the influence of other links among brain regions (Das et al., 2017). Therefore, it may be useful to combine our approach with other techniques for examining dynamic FC to gain a more complete view of the pathophysiology of neglect.

Overall, our results indicate that stroke leading to spatial neglect affects the temporal properties of functional interactions among large-scale networks, with a preferential configuration displaying low brain modularity and system segregation. In comparison to static functional connectivity studies, our findings offer two primary theoretical and clinical insights.

First, although in a small cohort of patients, the dynamic functional connectivity analyses identified a neglect-relevant brain state featuring widespread robust functional connections both within and between networks, with low modularity and system segregation. Notably, such brain configuration has not been described in previous studies employing static functional connectivity. Therefore, the dynamic interactions among brain systems might represent a key feature for higher functions such as spatial attention. Second, the temporal dynamics of neglect-relevant functional connectivity might guide protocol of non-invasive brain stimulation such as closed-loop, brain-state triggered TMS (Zrenner et al., 2016) for the treatment of spatial neglect.

## Limitations

The current study has several limitations. First, the relatively small sample size (*n* = 20). Nonetheless, the proportion of patients classified as neglect is consistent with previous reports and is representative of a clinical population of patients who had suffered from a right-hemisphere lesion. However, future studies are needed to confirm the sub-group analyses given that the computation of connectivity states was carried out only on 11 and 9 patients with and without neglect, respectively. Second, in contrast with other recent studies, we did not include dynamic FC data from healthy controls in our analyses. However, comparing patients with vs. without the deficit of interest (i.e., spatial impairment) is a suitable approach to identify and characterize brain states selectively associated with neglect beside the mere effect of the underlying structural lesions. Third, we investigated extrapersonal, egocentric neglect, assessed by cancellation tests. Future studies are needed to link brain states to different components of neglect, like, for instance, personal neglect. Finally, we employed a fixed-length sliding window approach to identify brain states. Methods based on modeling brain states, such as the Hidden Markov models, should be applied to further dissect latent brain states.

## Data availability statement

The raw data supporting the conclusions of this article will be made available by the authors, without undue reservation.

## Ethics statement

The studies involving human participants were reviewed and approved by Institutional Review Board (IRB) of IRCCS NEUROMED. The patients/participants provided their written informed consent to participate in this study.

## Author contributions

SS, FP, AD, and AB contributed to the study conception and design. LP and AB collected data. SS, AD, SLS, and AB analyzed data. SS and AB wrote the first draft of the manuscript. All authors commented on previous versions of the manuscript, read, and approved the final manuscript.
