# Supplementary material for: Dynamic brain states in spatial neglect after stroke
Source: Front Syst Neurosci. 2023 May 2;17:1163147. doi: 10.3389/fnsys.2023.1163147 (PMC10185806; doi:10.3389/fnsys.2023.1163147)
Supplement: Supplementary file 1 [file Table_1.DOCX]

Supplementary Table 1. List of 7 resting state networks (RSNs) and regions of interest (ROIs)

| **RSN** | **ROI** | **X** | **Y** | **Z** |
| --- | --- | --- | --- | --- |
| Visual | Occipital Medial | 2 | -79 | 12 |
| Visual | Occipital Medial | 0 | -93 | -4 |
| Visual | Occipital Lateral | -37 | -70 | 10 |
| Visual | Occipital Lateral | 38 | -72 | 13 |
| Dorsal Attention | Frontal Eye Field | -27 | -9 | 64 |
| Dorsal Attention | Frontal Eye Field | 30 | -6 | 64 |
| Dorsal Attention | Intraparietal Sulcus | -39 | -43 | 52 |
| Dorsal Attention | Intraparietal Sulcus | 39 | -42 | 54 |
| SensoriMotor | Lateral | -55 | -12 | 29 |
| SensoriMotor | Lateral | 56 | -10 | 29 |
| SensoriMotor | Superior | 0 | -31 | 67 |
| Cingulo-Opercular | Anterior Cingulate Cortex | 0 | 22 | 35 |
| Cingulo-Opercular | Anterior Insula | -44 | 13 | 1 |
| Cingulo-Opercular | Anterior Insula | 47 | 14 | 0 |
| Cingulo-Opercular | PreFrontal Cortex | -32 | 45 | 27 |
| Cingulo-Opercular | PreFrontal Cortex | 32 | 46 | 27 |
| Cingulo-Opercular | Supramarginal Gyrus | -60 | -39 | 31 |
| Cingulo-Opercular | Supramarginal Gyrus | 60 | -35 | 32 |
| Language | Inferior Frontal Gyrus | -51 | 26 | 2 |
| Language | Inferior Frontal Gyrus | 54 | 28 | 1 |
| Language | Posterior Superior Temporal Gyrus | -57 | -47 | 15 |
| Language | Posterior Superior Temporal Gyrus | 59 | -42 | 13 |
| Fronto-Parietal | Pre-Frontal Cortex | -43 | 33 | 28 |
| Fronto-Parietal | Posterior Parietal Cortex | -46 | -58 | 49 |
| Fronto-Parietal | Pre-Frontal Cortex | 41 | 38 | 30 |
| Fronto-Parietal | Posterior Parietal Cortex | 52 | -52 | 45 |
| Default Mode | Medial Pre-Frontal Cortex | 1 | 55 | -3 |
| Default Mode | Angular Gyrus | -39 | -77 | 33 |
| Default Mode | Angular Gyrus | 47 | -67 | 29 |
| Default Mode | Posterior Parietal Cortex | 1 | -61 | 38 |
